# Evaluation of fetal cell transplantation safety in treatment of diabetes: a three-year follow-up

**DOI:** 10.1186/s40200-014-0126-x

**Published:** 2015-04-22

**Authors:** Ensieh Nasli-Esfahani, Maryam Ghodsi, Peyvand Amini, Abbas Ali Keshtkar, Somayeh Amiri, Nika Mojahed-Yazdi, Ali Tootee, Bagher Larijani

**Affiliations:** Diabetes Research Center, Endocrinology and Metabolism Clinical Sciences Institute, Tehran University of Medical Sciences, Tehran, Iran; Endocrinology and Metabolism Research Center, Endocrinology and Metabolism Research Institute, Tehran University of Medical Sciences, Tehran, Iran; Endocrinology and Metabolism Research Center, Shari’ati Hospital, Tehran University of Medical Sciences, North Karegar St., 1411413137 Tehran, Iran

**Keywords:** Diabetes mellitus, Fetal stem cell, Cell therapy

## Abstract

**Background:**

Rapidly increasing prevalence of diabetes throughout the world highlights the importance of looking for new treatment options for the disease such as stem cell therapy. With regard to the increasing attention towards stem-cell therapy as a curative treatment for diabetes in recent years, it is of crucial importance to ensure the safety of this novel therapeutic technique. In this study we aim to evaluate the safety of fetal liver-derived cell suspension allotransplantation in the diabetic patients who had attended a clinical trial in 2007.

**Methods:**

44 out of a total number of 56 patients who had undergone either fetal liver-derived cell suspension allotransplantation or placebo injection in 2007 (IRCT number: 138811071414 N10) were contacted and recruited for the evaluation of possible complications. Patients were referred to a designated ophthalmologist and cardiologist to be screened for retinopathy and cardiovascular diseases. 24-hour urine was collected and tested for the evaluation of nephropathy; and, neuropathy was assessed by means of neuropathic symptoms and monofilament test.

**Results:**

There were no life-threatening complications nor significant differences in terms of evaluated diabetes complications ( retinopathy, neuropathy, nephropathy and cardiovascular diseases ) between the case and control groups. However, one case of meningioma was reported.

**Conclusions:**

Findings of our study demonstrated that stem cell transplantation can be considered a relatively safe procedure apart from one case of meningioma; it did neither cause any life-threatening complications nor increased the rate of the diabetes micro- and macrovascular complications.

## Introduction

Type 1 diabetes mellitus (T1DM) is demonstrated to be caused by an autoimmune mechanism which leads to destruction of β-cells in the pancreas [1]. Similarly, it is hypothesized that in type 2 diabetes mellitus (T2DM), β cells undergo apoptosis via some obscure immunological mechanisms. In both types of diabetes, the disease progress is towards reducing the number of islet cells due to immunological interactions [1-7] . Therefore, many novel therapeutic options have been developed for treatment of diabetes in recent years that focused on combining regenerative stimuli immunomodulation to preserve beta cell function [8].

Islet-cell transplantation and whole pancreas transplantation are considered as curative treatment options although their clinical benefits are limited. Scarcity of donors and the side effects of immunosuppressive drugs which are usually indicated after the transplantation can be considered as major limitations of these procedures. Therefore, there is a need to develop new therapeutic approaches for the treatment of diabetes such as stem-cell therapy as well as investigating their long-term safety [9-11]. Considering the novelty of the new approaches, there are limited data regarding their potential risks and complications. Therefore, it is of crucial importance to evaluate the long-term safety of such new therapeutic approaches.

Despite all advances in the field of stem cell therapy, there are still serious concerns remaining over the safety of this novel treatment [12]. In two different studies, two cases of benign tumors in the nervous system and the kidney are reported following stem-cell transplantation [13,14]. As stem-cell therapy is not widely applied and considering the novelty of the technique which makes it impossible to conduct extensive follow up studies [13,14], we aimed to conduct a 3-year safety follow-up of patients with diabetes who had undergone fetal liver-derived cell suspension allotransplantation in a study done by *Ghodsi et al.* in 2007 [15].

## Matherial and methods

56 patients with type one (n = 30) and type 2 (n = 26) diabetes who had previously participated in a double blind randomized controlled clinical trial in 2007 (Ethical Code: 0089 and IRCT number: 138811071414 N10), had been visited at the 6^th^ and the 12^th^ months after injection. Briefly, fetal liver-derived hematopoietic stem cells (HSCs) were isolated from legally and aborted human fetuses aged 6–12 weeks after obtaining an informed consent from the parents (mother or both of the parents). In order to determine chromosomal abnormalities and to identify the sex of the donated fetus, karyotyping was done for each fetal sample. Whole fetal liver was placed in Hank’s balanced salt solution without calcium and magnesium (HBSS, Sigma, USA) and dissociated and homogenized mechanically. The cell suspension was filtered through nylon mesh to undergo transplantation; and then, isolated cells were cryopreserved using 5% dimethyl sulfoxide (DMSO) in HBSS, (Wak Chemie, Germany) with a programmable freezer, and were transferred to liquid nitrogen for long term storage. Before transplantation, samples were thawed at 37oC and cryoprotectant was diluted by 5 milliliter normal saline before infusion. Total cell count in the prepared suspension was approximately 35-55 × 106, twenty percent of which was recognized as hematopoietic (CD34+) stem cells. The suspension was checked before, during, and after processing for aerobic, anaerobic and fungal contamination as well as viral infections. Rubella, Herpes Simplex Virus, Cytomegalovirus, Chlamydia, Mycoplasma Homonis, Toxoplasma Gondii and Treponema Pallidume were checked using ELISA (enzyme linked immunoassay). DNA/RNA extraction and polymerase chain reaction (real-time PCR) were done for checking viral contamination (HBV, HCV, and HIV). After evaluating the results, cell samples were known qualified for the transplantation. Injectable normal saline at the dosage of 5 ml was considered as the placebo solution. On the day of transplantation each participant in the intervention group received fetal liver-derived cell suspension at the dosage of approximately 35-55 × 106 cells (7-11 × 106 CD34+ HSCs) in 5 milliliter of normal saline intravenously. Participants in placebo group received 5 milliliter of normal saline intravenously [15].

Three years after the transplantation in 2009, the patients who were available through mailing address or telephone numbers were contacted and visited over a 3-months period. 44/56 signed an informed consent and agreed to participate in the current study. Unfortunately, 12/56 patients were not available at the address or telephone numbers they had given at the time of transplantation in 2007.

Patients were referred to the same ophthalmologist for the assessment of diabetes retinopathy, and similar to the previous visits [15], the results were reported as normal, Non–Proliferative Diabetic Retinopathy (NPDR), and Proliferative Diabetic Retinopathy (PDR). Peripheral arteries were examined through Posterior Tibialis and Dorsalis Pedis pulses . Similar to the previous study [15], neuropathy was defined by related symptoms complained by the patients. The same method was used for the assessment of neuropathy: patients were examined with 10-g monofilaments and the results were reported as presence or absence of neuropathy [16]. EMG and NCV were not performed. In our study, neuropathy was defined as burning pain, electrical or stabbing sensations, parasthesia, hyperesthesia, deep aching pain and the loss of 10-g monofilament perception at the distal halluces [16].

As for the assessment for nephropathy, 24-hours urine was collected and tested for proteinuria and the results were reported as no albuminuria, micro-albuminuria and macro-albuminuria [17,18]. For evaluating the cardiovascular complications, patients were referred to the same cardiologist. In addition to a complete history taking and thorough physical examination, ECG was performed in all patients and if necessary, exercise stress test and angiography were requested; and, the results were reported by the cardiologist as normal or Ischemic Heart Disease (IHD). History of myocardial infarction, unstable angina, positive exercise tolerance test, angiographic evidence of arterial stenosis, ECG abnormalities or the history of Coronary Artery Bypass Graft (CABG) were defined as IHD[19,20]. In addition, HbA1c levels had been measured to evaluate the glycemic control in these patients. All examiners were blinded in this study.

Statistical analysis was performed with the use of SPSS software (version: 17.0) and significance level was set at 0.05. Independent *t*-test and Chi square test (Fisher’s exact test) was used to compare complications between the intervention and placebo groups before and 3 years after transplantation.

## Results

In the 3^rd^ year of follow-up in 2009, 44 of 56 patients who had undergone fetal liver-derived cell suspension allotransplantation or received placebo injection were visited for the assessment of the course of their disease or any other potential life-threatening complications of the transplantation. The 12 missing patients whom we failed to follow because of the change in their contact phone number or mailing address consisted of both the intervention (one patient with T1DM and three patients with T2DM) and the placebo group (five patients with T1DM and three patients with T2DM) . The demographic data and HbA1c levels of all patients were recorded both at the baseline and 3 years post transplantation. The demographic data and HbA_1_c levels of those who were missed over the follow-up period were similar to that of those who could attend this study both in the intervention and placebo groups (Table 1).

**Table 1:** **Baseline demographic characteristics and HbA1c levels of the missing pati in the intervention and placebo groups**

	**Variable**	**Not missed**	**Missing group**	**p-value** ^**a**^
Intervention (N = 28)	Age (mean ± std)	34.95 ± 17.09	41.75 ± 15.88	0.465
	Female % (n)	14/24(58.3%)	4/4(100%)	0.107
	Duration of diabetes(mean ± std)	5.10 ± 2.62	5.75 ± 2.06	0.645
	HbA_1_c(%) at baseline	9.90 ± 1.79	11.30 ± 2.66	0.19
	HbA1c(%) at 1 year F.U	8.76 ± 1.81	9.00 ± 2.70	0.825
	variable	Not Missed	Missing Group	p-value^a^
		(N = 20)	(N = 8)	
Placebo (N = 28)	Age (mean ± std)	32.00 ± 14.09	24.25 ± 18.34	0.239
	Female % (n)	11/20(55.0%)	5/8(62.5%)	0.717
	Duration of diabetes(mean ± std)	5.47 ± 3.04	3.37 ± 2.98	0.109
	HbA1c(%) at baseline	9.78 ± 1.63	9.86 ± 0.96	0.895
	HbA1c(%) at 1 year F.U	8.31 ± 2.09	9.45 ± 2.32	0.253

^a^independent *t*-test.

In both type one and two diabetic patients, results of the clinical investigations three years after the procedure clearly revealed that there were no significant differences in the incidence or progression of diabetes micro vascular complications in patients who had either undergone fetal liver-derived cell suspension allotransplantation or received placebo compared to the baseline (Table 2).

**Table 2:** **Presence of retinopathy,neuropathy and microalbuminuria in patients with type 1 diabetes at the baseline and 3 years following the intervention**

**Type 1 diabetes**			**Retinopathy (baseline)**		**Total**	**P-Value**	**Rethinopathy (3rd year)**		**Total**	**P-Value**
			**Normal**	**PDR**			**Normal**	**NPDR**		
Groups	Intervention	Count	13	0	13	0.999^b^	11	1	12	0.999^b^
		(%)	100.0%	.0%	100.0%		91.7%	8.3%	100.0%	
	Placebo	Count	16	1	17		12	0	12	
		(%)	94.1%	5.9%	100.0%		100.0%	.0%	100.0%	
Total		Count	29	1	30		23	1	24	
		(%)	96.7%	3.3%	100.0%		95.8%	4.2%	100.0%	
			Neuropathy (Basal)		Total	P-Value	Neuropathy (3rd year)		Total	P-Value
			no_neurophathy	neuropathy+			no_neurophathy	neuropathy+		
Groups	Intervention	Count	12	1	13	0.433^b^	10	2	12	0.478^b^
		(%)	92.3%	7.7%	100.0%		83.3%	16.7%	100.0%	
	Placebo	Count	17	0	17		12	0	12	
		(%)	100.0%	.0%	100.0%		100.0%	.0%	100.0%	
	Total	Count	29	1	30		22	2	24	
		(%)	96.7%	3.3%	100.0%		91.7%	8.3%	100.0%	
			Microalbominuria (baseline)		Total	P-Value	Microalbominuria (3rd year)		Total	P-Value
			No Albominuria	Microalbominuria^+^			No Albominuria	Microalbominuria^+^		
Groups	intervention	Count	12	1	13	0.844^a^	12	0	12	0.307^a^
		(%)	92.3%	7.7%	100.0%		100.0%	.0%	100.0%	
	placebo	Count	16	1	17		11	1	12	
		(%)	94.1%	5.9%	100.0%		91.7%	8.3%	100.0%	
Total		Count	28	2	30		23	1	24	
		(%)	93.3%	6.7%	100.0%		95.8%	4.2%	100.0%	

^a^Pearson Chi-Square.

^b^Fisher’s Exact Test.

*Pearson Chi-Square is not computed because IHD (3rd year) is a constant.

PDR:Proliferative Diabetic Retinopathy, NPDR:None Proliferative Diabetic Retinopathy.

neuropathy+ : having neuropathy.

Microalbominuria+: 30 ≤ urine albominuria <300 (ng/mL).

IHD+: having Ischemic Heart Disease.

None of the patients with type 1 diabetes had cardiovascular complications, neither at the baseline nor after 3 years. For patients with type 2 diabetes, our findings showed that there was no statistically significant difference in terms of cardiovascular complications incidence in the intervention and placebo group after three years (Table 3).

**Table 3:** **Presence of retinopathy, neuropathy, microalbuminuria, and Ischemic Heart Disease(IHD) in patients with type 2 diabetes at the baseline and 3 years after the intervention**

**Type 2 diabetes**			**Retinopathy (baseline)**		**Total**	**P-Value**	**Rethinopathy (3rd year)**		**Total**	**P-Value**
			**Normal**	**PDR**			**Normal**	**NPDR**		
Groups	Intervention	Count	13	2	15	0.373^a^	12	0	12	0.400^b^
		(%)	86.7%	13.3%	100.0%		100.0%	.0%	100.0%	
	Placebo	Count	8	3	11		7	1	8	
		(%)	72.7%	27.3%	100.0%		87.5%	12.5%	100.0%	
Total		Count	21	5	26		19	1	20	
		(%)	80.8%	19.2%	100.0%		95.0%	5.0%	100.0%	
			Neuropathy (Basal)		Total	P-Value	Neuropathy (3rd year)		Total	P-Value
			no_neurophathy	neuropathy+			no_neurophathy	neuropathy+		
Groups	Intervention	Count	8	7	15	0.315^a^	8	4	12	0.292^a^
		(%)	53.3%	46.7%	100.0%		66.7%	33.3%	100.0%	
	Placebo	Count	8	3	11		7	1	8	
		(%)	72.7%	27.3%	100.0%		87.5%	12.5%	100.0%	
Total		Count	16	10	26		15	5	20	
		(%)	61.5%	38.5%	100.0%		75.0%	25.0%	100.0%	
			Microalbominuria (baseline)		Total	P-Value	Microalbominuria (3rd year)		Total	P-Value
			No Albominuria	Microalbominuria^+^			No Albominuria	Microalbominuria^+^		
Groups	intervention	Count	14	1	15	0.364^a^	11	1	12	0.999^b^
		(%)	93.3%	6.7%	100.0%		91.7%	8.3%	100.0%	
	placebo	Count	9	2	11		8	0	8	
		(%)	81.8%	18.2%	100.0%		100.0%	.0%	100.0%	
Total		Count	23	3	26		19	1	20	
		(%)	88.5%	11.5%	100.0%		95.0%	5.0%	100.0%	
			IHD (baseline)		Total	P-Value	IHD (3rd year)		Total	P-Value
			Normal	IHD +			Normal	IHD +		
Groups	intervention	Count	14	1	15	0.364^a^	9	3	12	0.494^a^
		(%)	93.3%	6.7%	100.0%		75.0%	25.0%	100.0%	
	placebo	Count	9	2	11		7	1	8	
		(%)	81.8%	18.2%	100.0%		87.5%	12.5%	100.0%	
Total		Count	23	3	26		16	4	20	
		(%)	88.5%	11.5%	100.0%		80.0%	20.0%	100.0%	

^a^Pearson Chi-Square.

^b^Fisher’s Exact Test.

PDR:Proliferative Diabetic Retinopathy, NPDR:None Proliferative Diabetic Retinopathy.

neuropathy^+^: having neuropathy.

Microalbominuria+: 30 ≤ urine albominuria <300 (ng/mL).

IHD+: having Ischemic Heart Disease.

However, the most important finding in the follow-up visits was the point that we found one patient who had developed meningioma probably with the origin of transplanted cells. This case is presented and discussed individually in another article.

## Discussion

Evaluating the safety of stem cell transplantation for treatment of diabetes, our results demonstrated that fetal liver-derived cell suspension allotransplantation did not lead to any life-threatening complications in the patients attending the follow-up visits three years after the transplantation in the three-year follow-up period. In addition, we observed no improvement or progress in diabetes micro- and macrovascular complications(retinopathy, neuropathy, and nephropathy and IHD) in the transplantation group compared with the placebo group as well as no new cases of the mentioned complications (Tables 2 and 3). Since the patients had received intensive diabetes care over the three years after the transplantation, it is hard to conclude that observing no progression in the noted diabetes complications was attributed whether to the cell therapy or resulted from diabetes care.

However, we noticed a case of meningioma in the transplantation group which will be discussed in detail in another article. Briefly, A 57-year-old female patient with type 1 diabetes who had undergone fetal liver-derived cell suspension allotransplantation, attended the clinic with the history of progressive bi-frontal headaches accompanied by nausea, vomiting, and visual disturbances over the past 8 months. Investigations revealed a 2-cm mass in the right temporal region. Thus, the patient underwent craniotomy and the lesion was removed and sent for pathological and genetic investigations. The results were suggestive of “Transitional Meningioma” with the origin of transplanted fetal hematopoietic stem cells.

In contrast to our results, different complications of stem-cell therapy are reported in several studies [13,14,21,22]. Voltarelli et al. reported one case of culture-negative bilateral pneumonia in addition to two cases of late endocrine dysfunction (hypothyroidism and hypogonadism) following autologous nonmyeloablative hematopoietic stem-cell transplantation in newly diagnosed type 1 diabetic patients [21,23]. However, it is still difficult to conclude that these complications developed as a result of stem cell transplantation only. Thus, the role of immunosuppressive drugs used in these studies should also be taken into consideration. We used fetal liver-derived stem-cells, which unlike hematopoietic stem-cells, do not need administration of cytotoxic drugs because of their low immunogenicity along with high self-renewal capacity [24]. Moreover, in a study by Amariglio et al., brain and spinal cord tumors were reported in a patient with ataxia telangiectasia who had undergone human fetal neural stem-cell transplantation [13]. However, these complications might be due to the direct injection of the cells into the target organ [13,14]. It is also suggested that complications observed in the study by Amariglio et al. are because of the increased susceptibility of developing tumors in patients with ataxia telangectasia which might be even more exaggerated after the injection of stem-cells [25]. Another case is reported in Taiwan in a patient with lupus nephritis who had undergone local renal injection of autologous hematopoietic stem cells collected from peripheral blood and mobilized by G-CSF. After the procedure, angiomyeloproliferative lesions were developed in the left kidney as well as smaller lesions in the right Adrenal gland and the liver. Similarly, it may be suggested that the tumors could have been developed due to the direct blind injection of stem cells into the kidney [13].

There were several limitations to our investigation. The most important one was the fact that we lost contact with some patients in the third year of follow-up because of the change in their addresses or phone numbers. Among the missing group (n = 12), 8 patients were in the placebo group and 4 patients (three patients with type 2 and one patient with type 1 diabetes) were in the transplantation group (Table 1). Therefore, it can be concluded from these data that the missing group might not have any significant impact on the final results of this study which aims to report the potential complications of stem cell transplantation for treatment of diabetes mellitus. Moreover, in terms of patient assessments, the methods through which the complications had been evaluated, should have been more accurate in preclinical assessments. Since the primary evaluations in 2007 had been mainly reported qualitatively rather than using quantitative scales, we had to use the same methods after 3 years in our follow up visits. In addition, as the major objective of this study was the evaluation of the incidence of malignancy following fetal HSCs transplantation, we did not use gold standard tests for the assessment of diabetes complications. Moreover, not all body systems have been assessed in this study for the presence of possible complications that is because such assessments were not performed at baseline, or their gold standard tests were invasive and costly. Therefore, we focused on the history and the symptoms expressed by the patients.

## Conclusion

To our knowledge, this is the first study that investigated the incidence of tumors and several other complications following fetal liver-derived cell suspension allotransplantation for the treatment of diabetes. The results of our study indicated that fetal stem cell transplantation did not cause any life-threatening complications as far as we observed and did not have any adverse effects on diabetes related complications. Taking the limitations of this study into consideration, it is still hard to express that observing no progression in diabetes complications is whether attributed to fetal stem cell transplantation or to the quality of diabetes care. Moreover, to definitely conclude that transplantation of fetal stem cells does not lead to any life-threatening events necessitates further studies with longer follow-up periods and more precise assessments using gold standard modalities in order to evaluate the safety and efficacy of this novel treatment option.
